# Influence of the length of target DNA overhang proximal to the array surface on discrimination of single-base mismatches on a 25-mer oligonucleotide array

**DOI:** 10.1186/1756-0500-7-251

**Published:** 2014-04-17

**Authors:** Jenny Tomlinson, Catherine Harrison, Neil Boonham, Sarah A Goodchild, Simon A Weller

**Affiliations:** 1 The Food and Environment Research Agency, Sand Hutton, YO41 1LZ York, UK; 2 Defence Science and Technology Laboratory, Porton Down, SP4 0JQ Salisbury, UK

**Keywords:** Oligonucleotide microarray, Mismatch discrimination, SNP

## Abstract

**Background:**

The performance of probes on an oligonucleotide microarray can be characterised in terms of hybridisation signal strength and the ability to discriminate sequence mismatches between the probe and the hybridising target strand, such as those resulting from SNPs. Various properties of the probe affect mismatch discrimination, such as probe length and the position of mismatched bases, and the effects of these factors have been well characterised in a variety of array formats.

**Results:**

A low-density microarray was developed to systematically investigate the effect of a probe’s position within hybridised target PCR products on the tolerance and discrimination of single-nucleotide mismatches between the probe and target. In line with previous reports, hybridisation signals were attenuated by different degrees depending on the identity of the mismatch, the position of the mismatch within the probe, and the length of the PCR product. However, the same mismatch caused different degrees of attenuation depending on the position of the probe within the hybridising product, such that improved mismatch discrimination was observed for PCR products where a greater proportion of the total length was proximal to the array surface.

**Conclusions:**

These results suggest that the degree of mismatch discrimination can be influenced by the choice of PCR primers, providing a means by which array performance could be fine-tuned in addition to manipulation of the properties of the probes themselves.

## Background

High-density microarrays in a conventional glass slide format with fluorescence detection, such as those used for gene expression studies, pose a relatively high operational burden due to time-consuming protocols and the cost of the equipment required for high-resolution fluorescence detection. In the context of pathogen detection, however, the requirement is more likely to be for a method which allows large numbers of samples to be tested efficiently at one time. Low-density microarrays, such as those using the ArrayTube (AT) platform [1-3] allow the detection of a smaller number of targets (typically < 100) with improved simplicity of handling and low-cost scanning instrumentation.

A typical low-density array protocol involves subjecting sample DNA to multiplex PCR with labelled nucleotides or primers, followed by application of the labelled products to the array and identification of the probes to which the products have hybridised. A frequently used approach is to select conserved target genes, enabling amplification using broad-range PCR primers followed by discrimination of different products by hybridisation to specific probes [4]. Conversely, in order to reliably detect sequence variants (for example, different viral strains or novel viruses) it can be necessary to tolerate sequence differences both in the PCR primers and the probes on the array [5]. In order to detect diverse targets (for example, bacterial and viral pathogens) on a single array, it may therefore be necessary to maximise mismatch discrimination for some targets while tolerating mismatches for others.

The performance of probes on an array is affected by factors including probe length [6,7], number and distribution of mismatches between the probe and target [8-10], orientation of attachment to the array surface and presence or absence of spacers [11,12]. The effects of mismatched bases on probe-target hybridisation have been studied in great detail, using microarrays in a variety of formats [10,13]. Greater specificity is achieved by using shorter probes [7], but longer probes are associated with higher hybridisation signals [6]. Furthermore, mismatches located at the centre of the probe have a greater effect on hybridisation signals than mismatches close to the ends of the probe [8,13,14]. Hybridisation is also dependent on the properties of the target nucleic acid; in particular, relatively long targets are associated with secondary structure which can significantly hinder hybridisation [15-17]. The performance of the array is therefore also influenced by the PCR primers used, in terms of the specificity they confer, their general suitability for PCR (since array sensitivity is largely determined by the sensitivity of the PCR) and the secondary structure of the amplified product. Generalisations can be made about favourable properties of the PCR products, such as length [10] and labelling method, for optimal performance of an array in terms of signal strength, specificity and limit of detection. The location of the PCR primers within the target sequence will also determine the position of the probe within the amplified product. Peytavi et al. [18] reported that the position of the probe can affect hybridisation signal strength; while Stedtfeld at al [19] found that false positive signals can be caused by interaction between overhanging target nucleic acid and labelled background DNA. We hypothesise that the position of the probe within the PCR product (i.e. it’s proximity to the 5′ or 3′ end of the hybridising strand) could also influence its performance in terms of the tolerance or discrimination of mismatches. A 25-mer oligonucleotide array in an AT format was designed to investigate this hypothesis.

## Methods

### Probe design

Perfect match probes with a length of 25 bases and a melting temperature of approx. 62°C were designed to target *Burkholderia pseudomallei* chromosome II (accession number CP000571). They were selected in regions of the sequence (Table 1) such that single-base changes could be introduced to result in eight mismatches between the probe and the target sequence (AA, AG, GG, CC, AC, CT, TT, and GT) at three different positions (in the 3′ third, the middle third, and the 5′ third). In this way, sets of 25 probes were constructed: a perfect match probe and 24 mismatch probes each containing a single mismatch with the target sequence. Microarrays were manufactured by Alere (Jena, Germany) using their ArrayTube platform and consisted of oligonucleotide probes with a 3′ amino modification and C6 spacer. Probes were spotted in duplicate.

**Table 1:** Probe sequences

**Probe name**	**Sequence (5′ to 3′)**
PM 1	ACTACGACACACATGACATGATCAA
5AA 1	AC**A**ACGACACACATGACATGATCAA
MAA 1	ACTACGACACACA**A**GACATGATCAA
3AA 1	ACTACGACACACATGACATGA**A**CAA
5AG 1	ACTA**A**GACACACATGACATGATCAA
MAG 1	ACTACGACACA**A**ATGACATGATCAA
3AG 1	ACTACGACACACATGACATGAT**A**AA
5GG 1	ACTA**G**GACACACATGACATGATCAA
MGG 1	ACTACGACACA**G**ATGACATGATCAA
3GG 1	ACTACGACACACATGACATGAT**G**AA
5CC 1	ACTAC**C**ACACACATGACATGATCAA
MCC 1	ACTACGACACACAT**C**ACATGATCAA
3CC 1	ACTACGACACACATGACAT**C**ATCAA
5 AC 1	ACTAC**A**ACACACATGACATGATCAA
MAC 1	ACTACGACACACAT**A**ACATGATCAA
3 AC 1	ACTACGACACACATGACAT**A**ATCAA
5CT 1	ACTAC**T**ACACACATGACATGATCAA
MCT 1	ACTACGACACACAT**T**ACATGATCAA
3CT 1	ACTACGACACACATGACAT**T**ATCAA
5TT 1	ACT**T**CGACACACATGACATGATCAA
MTT 1	ACTACGACACAC**T**TGACATGATCAA
3TT 1	ACTACGACACACATGACATG**T**TCAA
5GT 1	ACT**G**CGACACACATGACATGATCAA
MGT 1	ACTACGACACAC**G**TGACATGATCAA
3GT 1	ACTACGACACACATGACATG**G**TCAA
PM 2	ACGATATCCTCGAAAAGACGATCAA
5AA 2	ACGA**A**ATCCTCGAAAAGACGATCAA
MAA 2	ACGATATCC**A**CGAAAAGACGATCAA
3AA 2	ACGATATCCTCGAAAAGACGA**A**CAA
5AG 2	A**A**GATATCCTCGAAAAGACGATCAA
MAG 2	ACGATATCCT**A**GAAAAGACGATCAA
3AG 2	ACGATATCCTCGAAAAGACGAT**A**AA
5GG 2	A**G**GATATCCTCGAAAAGACGATCAA
MGG 2	ACGATATCCT**G**GAAAAGACGATCAA
3GG 2	ACGATATCCTCGAAAAGACGAT**G**AA
5CC 2	AC**C**ATATCCTCGAAAAGACGATCAA
MCC 2	ACGATATCCTC**C**AAAAGACGATCAA
3CC 2	ACGATATCCTCGAAAAGAC**C**ATCAA
5 AC 2	AC**A**ATATCCTCGAAAAGACGATCAA
MAC 2	ACGATATCCTC**A**AAAAGACGATCAA
3 AC 2	ACGATATCCTCGAAAAGAC**A**ATCAA
5CT 2	AC**T**ATATCCTCGAAAAGACGATCAA
MCT 2	ACGATATCCTC**T**AAAAGACGATCAA
3CT 2	ACGATATCCTCGAAAAGAC**T**ATCAA
5TT 2	ACG**T**TATCCTCGAAAAGACGATCAA
MTT 2	ACGATATCCTCG**T**AAAGACGATCAA
3TT 2	ACGATATCCTCGAAAAGACG**T**TCAA
5GT 2	ACG**G**TATCCTCGAAAAGACGATCAA
MGT 2	ACGATATCCTCG**G**AAAGACGATCAA
3GT 2	ACGATATCCTCGAAAAGACG**G**TCAA

Mismatched bases are indicated in bold.

### PCR primer design

**Figure 1:**
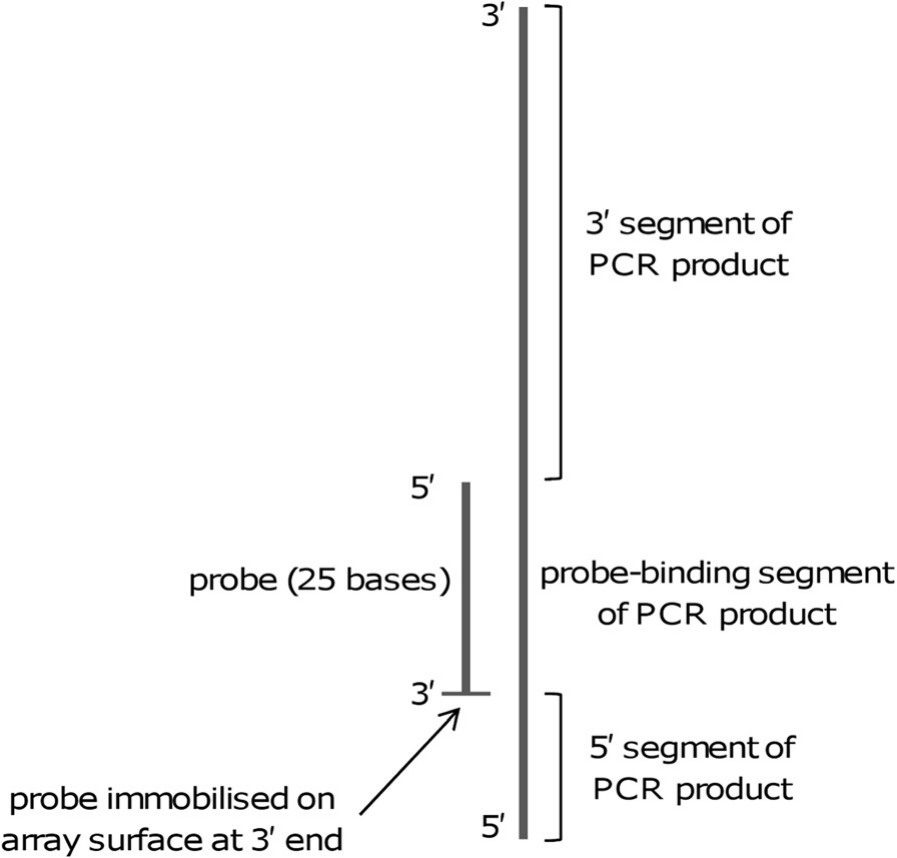
**Illustration of the position of probes within PCR-amplified product.** The section of the product overhanging the probe proximal to the array surface is referred to as the 5′ segment of the product, and the section overhanging the 5′ end of the probe is referred to as the 3′ segment of the product.

PCR primers were designed based on *B. pseudomallei* sequence (accession number CP000571) to generate PCR products of different lengths and in different positions relative to the probes. The PCR products generated by different primer pairs could be characterised in terms of the total length of the PCR product and the length of the section of the PCR product overhanging the probe sequence at its 3′ end, referred to as the 5′ segment of the product (see Figure 1); this is the region referred to as the ‘surface-proximal tail’ by Stedtfeld et al. [19]. Primers were synthesised by MWG Eurofins (Ebersberg, Germany). Their sequences are shown in Table 2.

**Table 2:** Primers used for biotin-labelled PCR

**Forward primer**	**Forward primer sequence (5′ to 3′)**	**Reverse primer**	**Reverse primer sequence (5′ to 3′)**	**Amplicon length (bases)**	**Length of 5′ segment (bases)**
for1	CTGGAGGAGCCTTCCCTAC	rev1	GTCGCGACTGCCGCTC	94	43
for2	CCTCGCATTGCTCAAGCACA	rev1	as above	145	43
for3	CGCGACACGCCCATGT	rev1	as above	243	43
for4	GCGGACATCGACGGCTATGT	rev1	as above	312	43
for4 lim*	AACGCGCGGACATCGACGGCTATGT	rev1	as above	317	43
for5	GCGCATACGATTCTTCAAAAGGC	rev1	as above	978	43
for3	as above	rev2	ACGCCTTACGTATCGGATCG	298	107
for6	CTTCGGGCCACCGCTACTA	rev3	TGTGCTGTGGGCCGTGCT	300	149
for1	as above	rev4	TCACCGCGCCTTGCTCGT	242	191
for2	as above	rev4	as above	293	191
for3	as above	rev4	as above	382	191
for7	as above	rev4	as above	624	191
for5	as above	rev4	as above	1126	191
for1	as above	rev5	CCTCGGGCGTTTCGATCAC	323	272
for4	as above	rev5	as above	541	272
for8	as above	rev5	as above	878	272
for5	as above	rev5	as above	1027	272
for8	CCAATGCATGTCGGCTCGC	rev6	ACACCTCTTGCACGGAACCG	296	23**
for9	GACACAAGCCGCGAACTGAC	rev7	CCCAATGTCCGACATAGCC	295	95**
for10	GAGCATCTTCGCGCCATAG	rev8	TGTCGCGCTCGTTCGCTG	289	149**
for7	CCTTTGCCGTCAGCTTCCG	rev9	TAGTAGCGGTGGCCCGAAG	301	201**
for11	CAGTTGTCCCTGAAGCGCCT	rev10	TTGAGCAATGCGAGGCTGC	317	246**

*Modified for asymmetrical (Linear-After-The-Exponential) PCR.

**Probe set 2 – all other primer combinations for probe set 1.

### DNA extraction

*Burkholderia pseudomallei* CLO2 DNA was prepared and quanitified by the Defence Science and Technology Organisation (DSTO) in Melbourne, Australia. The DNA extract was sterility checked to enable work under Biological Safety Level 2 (BSL2) conditions.

### PCR with biotin labelling

PCR was carried out in 50 μl reactions containing 1× GoTaq Flexi Colourless Buffer (Promega, Madison, WI, USA); 1.5 mM MgCl_2_; 80 μM each dATP, dCTP and dGTP; 52 μM dTTP; 28 μM biotin-11-dUTP (Thermo Scientific, Waltham, MA, USA); 300 nM forward primer; 300 nM reverse primer; 1.25 units Go Taq polymerase (Promega) and 1 ng *B. pseudomallei* DNA. Primer combinations were as shown in Table 2. Cycling conditions were 95°C for 2 min, followed by 30 three-step cycles of 95°C for 30 s 58°C for 30 s and 72°C for 1 min, with a final extension step at 72°C for 5 min. After amplification, reactions were purified using the QIAquick PCR Purification kit (Qiagen, Hilden, Germany) according to the manufacturer’s instructions and eluted in nuclease-free water. Approximate DNA concentrations of the purified PCR products were determined using a Qubit fluorometer and dsDNA HS Assay Kit (Life Technologies, Foster City, CA, USA) according to the manufacturer’s instructions. Approximate copy numbers were estimated on the basis of DNA concentration and the molecular weight of the predicted product. Each primer combination was initially tested in unlabelled PCR with dNTPs at 80 μM each and the products analysed by agarose gel electrophoresis to confirm the approximate size of the product and absence of primer dimers or other amplification artifacts (data not shown).

### Asymmetric PCR with biotin labelling

Asymmetric PCR is often used to generate predominantly single-stranded PCR products for hybridisation to microarrays, and the presence of the complementary, non-hybridising strand has been shown to influence hybridisation efficiency and other factors [18,20]. In order to investigate the effect on tolerance and discrimination of mismatches, forward primer for4 was modified for asymmetric PCR taking into account design considerations for Linear-After-The-Exponential (LATE) PCR [21] as shown in Table 2. PCR reactions and cycling conditions were the same as those used for symmetric PCR, except that the concentration of the reverse (excess) primer was increased to 500 nM and the concentration of the modified forward (limiting) primer was reduced to 25, 50 or 100 nM to give primer ratios of 20:1, 10:1 and 5:1, respectively. Approximate total DNA concentrations (single- and double-stranded DNA) were determined for symmetrical and asymmetrical PCR products using a Qubit fluorometer and ssDNA Assay Kit (Life Technologies) according to the manufacturer’s instructions, and concentrations were adjusted to give approximately equal total DNA concentrations. The symmetrical and asymmetrical PCR products were treated with S1 nuclease and subjected to agarose gel electrophoresis to determine the presence or absence of single-stranded amplification products [22]. For treatment with S1 nuclease, 10 μl PCR product was combined with 20 units S1 nuclease and 4 μl 5× S1 nuclease reaction buffer (Thermo Scientific) in a final reaction volume of 20 μl and incubated at 37°C for 30 min. Treated and untreated PCR products (10 μl and 5 μl, respectively) were analysed by electrophoresis in 1.2% agarose gels containing GelRed nucleic acid stain (Biotium, Hayward, CA) at a final concentration of 0.5×.

### Array hybridisation and analysis

Biotin-labelled PCR products were made up to a volume of 40 μl in nuclease-free water and mixed with 70 μl Nexterion Hybridisation Buffer (Schott, Mainz, Germany), then heated to 95°C for 4 min and cooled to 55°C. ArrayTubes were conditioned by applying 500 μl nuclease-free water and incubating at 55°C with shaking at approx. 500 rpm for 5 min, after which the water was removed and 500 μl Nexterion Hybridisation Buffer was added and incubated at 55°C for 5 min with shaking. The buffer was removed and the pre-heated PCR products were applied to the ArrayTubes and incubated at 55°C for 1 h with shaking at approx. 500 rpm. The ArrayTubes were washed three times: first in 2× SSC containing 0.01% Triton X-100 (Sigma-Aldrich, St Louis, MO, USA), then 2× SSC and finally in 0.2× SSC; for each wash step, the previous solution was removed, 500 μl relevant wash buffer was added and the ArrayTubes were incubated for 5 min at room temperature with shaking at approx. 500 rpm. The final wash buffer was removed and 100 μl blocking solution containing 1× phosphate buffered saline (PBS), 2% dried milk powder and 0.05% Triton X-100 was applied to each ArrayTube and incubated at 30°C for 15 min, then removed. HRP-linked anti-biotin antibody (New England Biolabs, Ipswich, MA, USA) was diluted 1 in 100 in blocking solution and 100 μl diluted antibody was applied to each array and incubated for 15 min at room temperature. Finally, the ArrayTubes were washed three times as described above in 1x PBS containing 0.1% Tween 20 (Sigma) (500 μl for each wash step). The final wash buffer was removed and 100 μl SeramunGrün Chip substrate (Seramun Diagnostica, Wolzig, Germany) was added to each ArrayTube. The ArrayTubes were scanned after approx. 15 – 20 min using an ATR-03 ArrayTube Reader (Alere), and images were analysed using the Iconoclust software package (Alere) as described by Cannon et al. [1].

## Results

### Effect of probe position on single base mismatch discrimination

**Figure 2:**
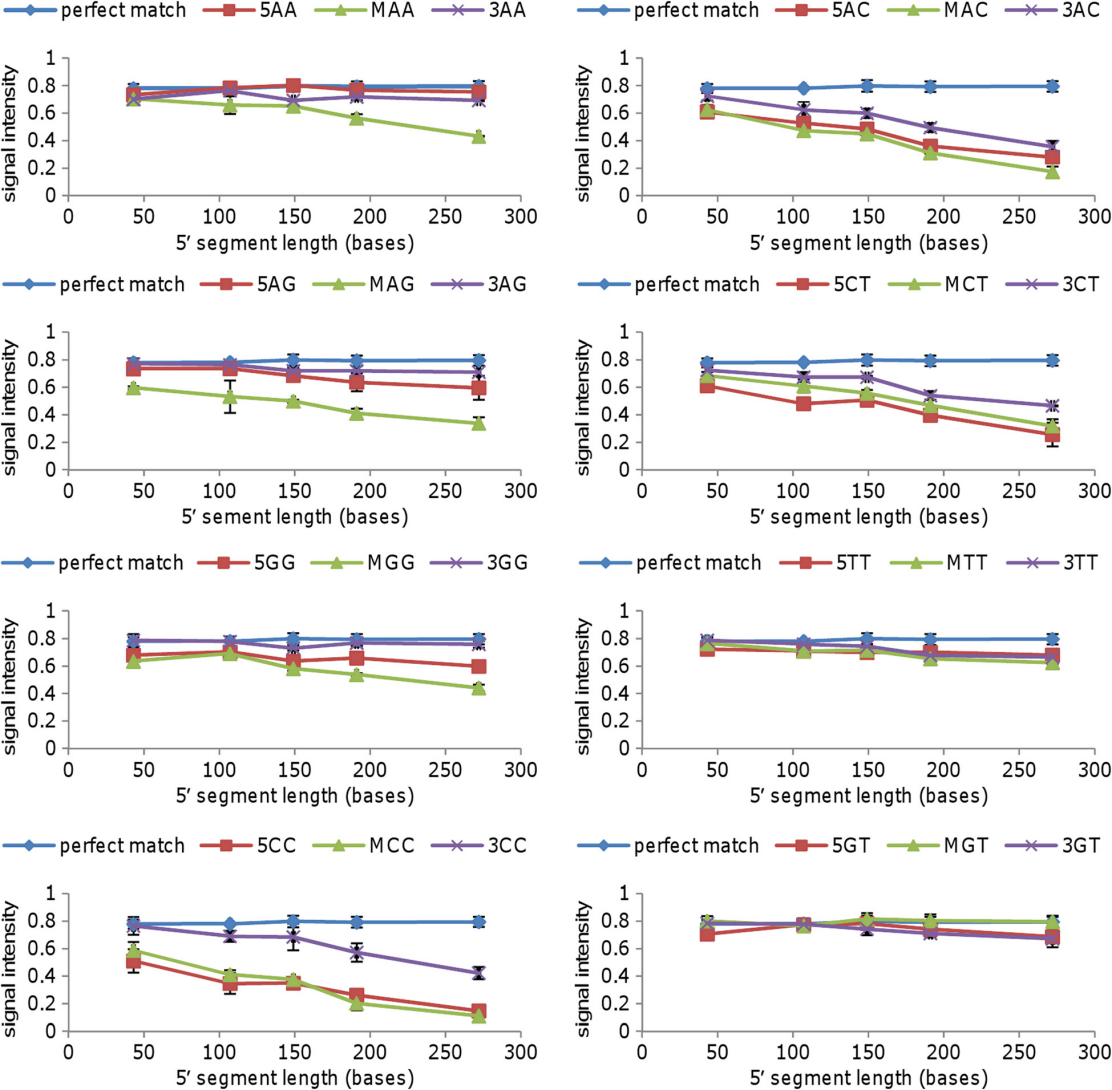
**Array analysis of PCR products with similar overall length but different 5′ segment lengths: probe set 1.** Panels show the signal intensity for perfect match and mismatch probes hybridised to PCR products with total lengths between 293 and 323 bases and 5′ segment lengths ranging from 43 to 272 bases; each panel shows the effect of a different mismatch at three positions within the probe. Approximately 10^12^ copies of PCR amplicon were applied to each array. Results shown are mean values for duplicate spots on the same array (error bars show standard deviation). 5AA = AA mismatch between probe and PCR product located in the 5′ third of the probe, etc.

**Figure 3:**
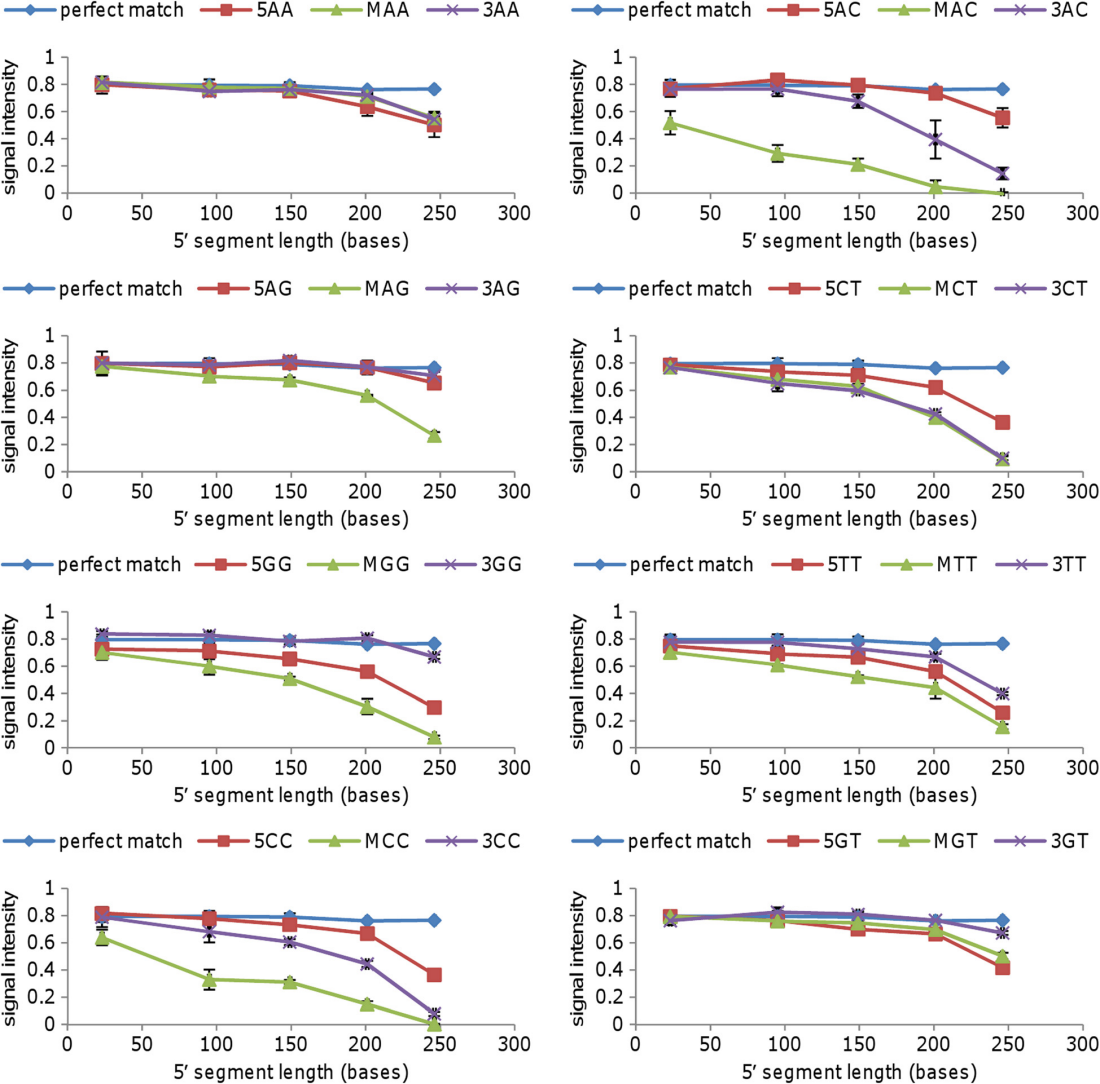
**Array analysis of PCR products with similar overall length but different 5′ segment lengths: probe set 2.** Panels show the signal intensity for perfect match and mismatch probes hybridised to PCR products with total lengths between 289 and 317 bases and 5′ segment lengths ranging from 23 to 246 bases; each panel shows the effect of a different mismatch at three positions within the probe. Approximately 10^12^ copies of PCR amplicon were applied to each array. Results shown are mean values for duplicate spots on the same array (error bars show standard deviation). 5AA = AA mismatch between probe and PCR product located in the 5′ third of the probe, etc.

Figures 2 and 3 shows typical results for arrays hybridised with PCR products (approximately 10^12^ copies per array) of similar total length (289 to 323 bases) but with 5′ segments ranging in length from 23 to 272 bases. Results are shown for eight different mismatches (AA, AC, AG, CT, GG, TT, CC and GT) in three different positions in the probe, as shown in Table 1. Similar signal intensities were observed for hybridisation to the perfect match probe, regardless of the probe position. Signal attenuation was observed for some mismatches in both probe sets (for example, AC and CC), while a GT mismatch was tolerated in both sets, and other mismatches (for example, AA and TT) were tolerated in the sequence context of one set but not the other. Mismatches located in the central section of the probe typically resulted in greater signal attentuation than equivalent mismatches located closer to the 3′ or 5′ end of the probe, in common with the results of others using various microarray platforms [7,8,14]. However, as shown in Figures 2 and 3, the degree of attenuation was observed to increase as the length of the 5′ segment of the PCR product (proximal to the array surface) was increased.

### Effect of probe position on limit of detection

PCR products with similar total lengths but different 5′ segment lengths were diluted and hybridised to arrays to determine the limit of detection for hybridisation to perfect match probes. No difference in limit of detection was observed for a pair of PCR products with 5′ segment lengths of 43 and 272 hybridised to perfect match probe 1, or for PCR products with 5′ segment lengths of 23 and 246 hybridised to perfect match probe 2, as shown in Table 3.

**Table 3:** Limit of detection of PCR products with similar total lengths but different 5′ segment lengths

		Signal intensity (mean ± sd)*		
**Total length (bases)**	**5′ segment length (bases)**	**Approx. 8 × 10**^**9 **^**copies**	**Approx. 8 × 10**^**8 **^**copies**	**Approx. 8 × 10**^**7 **^**copies**
312	43	0.63 ± 0.03	0.23 ± 0.03	-
323	272	0.62 ± 0.01	0.22 ± 0.02	-
296	23	0.66 ± 0.06	0.28 ± 0.05	-
317	246	0.57 ± 0.03	0.24 ± 0.00	-

*Results shown are mean values for duplicate spots on the same array; -: negative.

### Combined effect of total product length and probe position

**Figure 4:**
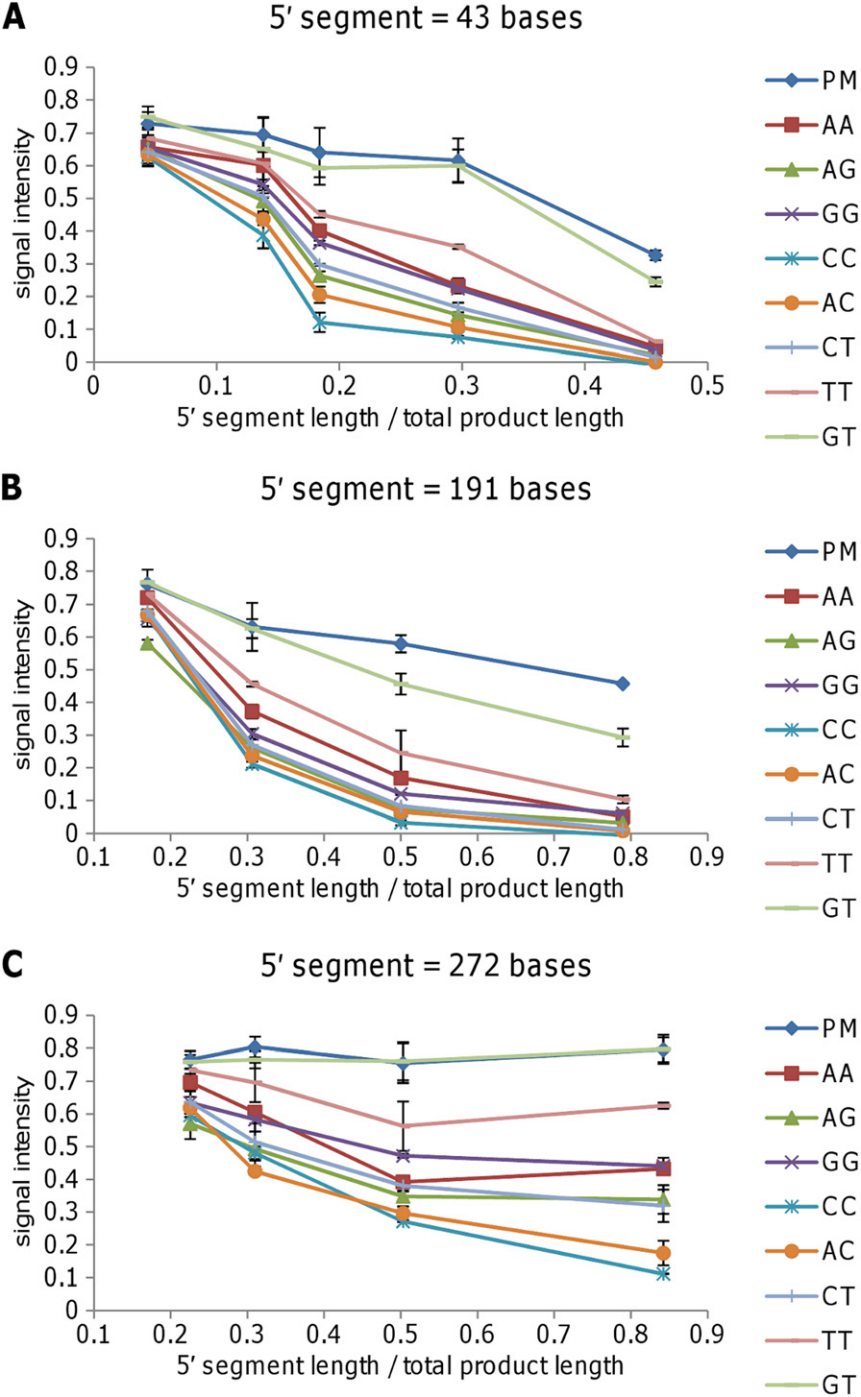
**Array analysis of PCR products with the same 5′ segment length but different total lengths.** Arrays were hybridised with PCR products with constant 5′ segment lengths (**A**: 43 bases; **B**: 191 bases; **C**: 272 bases) but different overall lengths ranging from 94 to 978 bases, 242 to 1126 bases, and 323 to 1207 bases, respectively. Signal intensities were normalised relative to the signal for the perfect match probe on each array and are plotted against the 5′ segment length shown as a proportion of the total product length. Approximately 10^12^ copies of PCR amplicon were applied to each array. Results shown are mean values for duplicate spots on the same array (error bars show standard deviation). PM = perfect match probe. AA = AA mismatch between probe and PCR product, etc.

Figure 4 shows typical results for groups of arrays hybridised with PCR products with the same 5′ segment length (43, 191 or 272 bases) and total lengths ranging from 94 to 978, 242 to 1126 or 323 to 1207 bases, respectively. Note that results are shown only for mismatches located in the middle third of the probes. As in the previous experiment, a GT mismatch was tolerated while other mismatches caused a greater degree of signal attenuation. The greatest signal attenuation was observed for PCR products where the surface-proximal 5′ segment constituted a larger proportion of the total PCR products; single base mismatches had a greater effect on signal intensity for short PCR products with a proportionally longer 5′ segment.

### Effect of primer ratio

**Figure 5:**
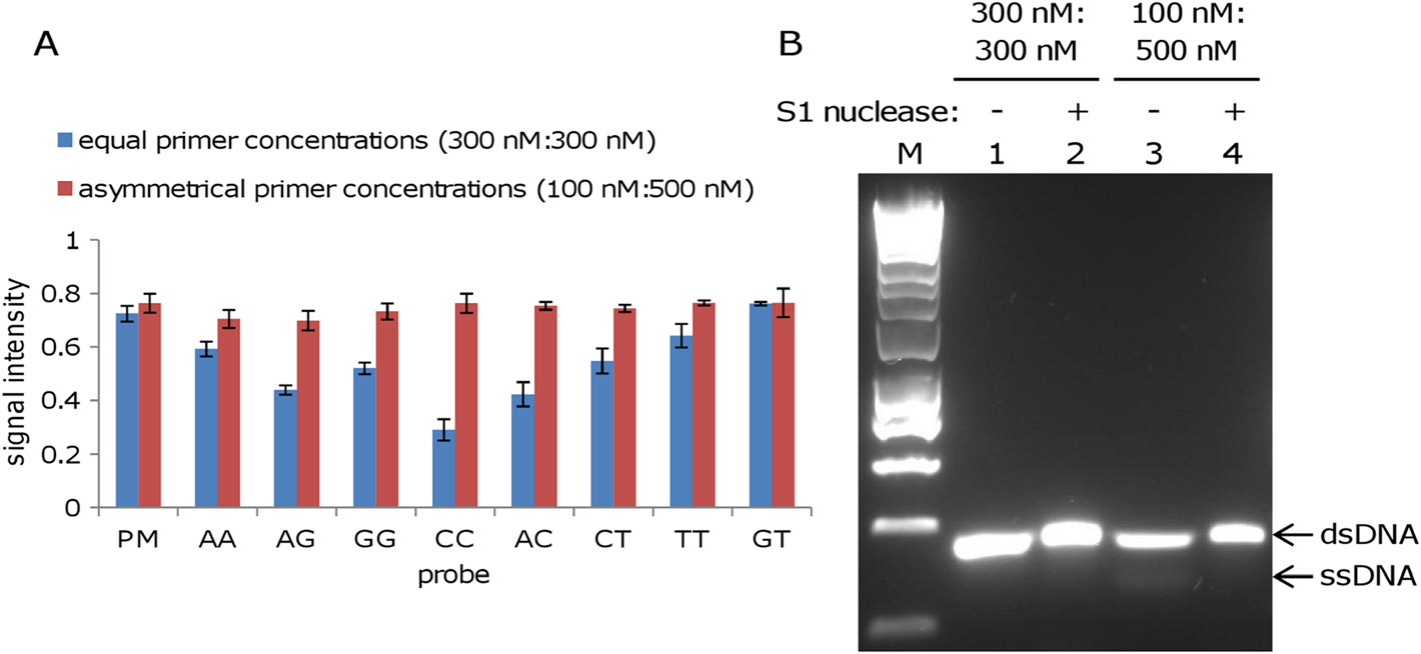
**Array analysis of symmetrical and asymmetrical PCR products. A.** Signal intensity for hybridisation of symmetrical and asymmetrical PCR products of 312 and 317 bases, respectively, both with a 5′ segment length of 43 bases. Asymmetrical PCR was carried out using a forward primer modified for linear-after-the-exponential (LATE) PCR, with a primer ratio of 1:10. Approximately 500 ng total DNA (single-stranded plus double-stranded) was hybridised to each array. Results shown are mean values for duplicate spots on the same array. AA = AA mismatch between probe and PCR product, etc. **B.** Agarose gel electrophoresis of symmetrical and asymmetrical PCR products. The products of symmetrical and asymmetrical PCR were visualised by agarose gel electrophoresis with GelRed nucleic acid stain before and after treatment with S1 nuclease to confirm that the product of asymmetrical PCR contained single-stranded DNA (removed by S1 nuclease) as well as double-stranded DNA.

Figure 5A shows results for arrays hybridised with products of symmetrical PCR and asymmetrical PCR (primer ratio 1:5); similar results were observed for PCR products obtained using primer ratios of 1:10 and 1:20 (data not shown). Note that results are shown only for mismatches located in the middle third of the probes. The symmetrical and asymmetrical PCR products had total lengths of 312 and 317 bases, respectively, and 5′ segment lengths of 43 bases. Figure 5B shows the symmetrical and asymmetrical PCR products analysed by agarose gel electrophoresis before and after treatment with S1 nuclease to demonstrate the presence of single-stranded DNA in the product generated by asymmetrical PCR. For the product of symmetrical PCR, signal attenuation was observed for some mismatches; however, similar signals were observed for the perfect match probe and all mismatched probes for the asymmetrical product.

## Discussion

The ArrayTube hybridisation architecture in the format used here contains around 200 locations for the deposition of DNA probes depending on the spacing between spots. With two replicates of each probe and an appropriate multiplexed PCR this allows the detection/discrimination of around 80–90 targets and subtyping of panels of associated agents along with appropriate controls such as for DNA extraction. Since this is an open platform, the user has control over the number of targets and the specific properties of the probes being used. The technology can also be used in a strip format for the simultaneous processing of up to 96 arrays, potentially allowing the development of higher throughput screening capabilities than other multiplex PCR-based platforms [23].

Clinical diagnostic applications require methods with high sensitivity and robustness. By using an array format it is possible to screen a sample for multiple targets from the same agent (for example, plasmid and chromosomal sequences) [3,24] and this redundancy has the potential to increase the robustness of detection in terms of the avoidance of both false positives and false negatives [24]. The ability to discriminate small sequence differences is crucial for some purposes, such as the discrimination of SNPs in the identification of antimicrobial resistance [25] or differentiation of bacterial genotypes [26]. Microarrays for pathogen detection are often designed to detect a relatively narrowly defined group of targets (for example, a particular bacterial taxon in a specified clinical matrix). More versatile arrays may be developed to perform a set of related functions, such as detection of a range of organisms and identification of the presence of antimicrobial resistance markers [4], or simultaneous detection of diverse agents, for example, bacteria and viruses, in the same sample. For the effective detection of viruses it may be necessary to tolerate multiple sequence differences, while for the detection of bacteria there may be a need to accurately discriminate between targets on the basis of minor sequence differences. While sensitivity will be largely influenced by PCR efficiency, our results indicate that it could be possible to leverage the specificity (or conversely, inclusivity) of detection by manipulating the length of the target’s PCR amplicon, positioning the probe with respect to the 5′ end of the hybridising strand and changing the primer ratio to generate double-stranded or partially single-stranded products.

In line with previous reports, we observed that different mismatches were tolerated to different degrees, with the greatest effect when the mismatch was located close to the centre of the probe [8,27,28]. In the limited probe sets that we used, a GT mismatch was tolerated (little or no attenuation of the hybridisation signal) while CC, AC and CT mismatches caused significant signal attenuation. However, we also observed that the same mismatched probe was observed to give different signal intensities when approximately equal numbers of copies of PCR products differing in terms of probe position were hybridised to the array. The maximum effect of single mismatches (and in particular CC, AC and CT mismatches) was observed for PCR products where a greater proportion of the total length was at the 5′ end relative to the probe (and therefore proximal to the array surface). A reduction in the discrimination of individual mismatches was observed if PCR primers were used at unequal concentrations resulting in generation of some single-stranded product, presumably because the complementary non-target strand competes with the shorter mismatched probe for hybridisation to the target strand. This is consistent with the proposal of Peytavi et al. [18] that the complementary strand destabilises the target-probe duplex, particularly in relation to the length of the distal segment of the hybridised target. We did not observe a reduction in signal strength with a longer 3′ segment of the PCR product (distal to the array surface) as was reported by Peytavi et al. (in fact, as shown in Figure 4, signal strength decreased with a shorter 3′ segment length); however, there are differences between the two experimental systems, including the probe orientation and hybridisation conditions, which could partially explain this difference. Our results suggest that in the experimental set-up used here, mismatch discrimination is most likely to be achieved using equal primer concentrations, while inclusive detection of sequence variants would be enhanced by the use of asymmetrical primer concentrations.

It should be noted that the results presented are for PCR using single primer pairs, and factors such as product length and primer ratio may impact on the performance of a primer pair in a multiplex reaction in terms of sensitivity. For example, shorter PCR amplicons may generally be preferable due to the likelihood of higher yields [29] and reduced formation of intra- and intermolecular structures which are reported to hinder hybridisation [15,18,19].

## Conclusions

Our results suggest a strategy for the rational design of PCR primers in order to leverage the exclusivity (specificity) or inclusivity of detection of individual targets on the array. Specifically, positioning a probe within the PCR product such that there is a longer section proximal to the array surface may increase the discrimination of single mismatches. As these primer design strategies could be applied independently to each target as required, this presents a means of fine-tuning an array for detection of potentially diverse targets. Optimisation of array performance by adjustment of the primers used for multiplex PCR has the potential to increase flexibility and cost effectiveness in comparison with redesigning the probes themselves.

## Competing interests

The authors declare that they have no competing interests.

## Authors’ contributions

JT, SAW, NB, SAG conceived and designed the experiments; JT, CH performed the experiments and analyzed the data. JT, SAW, NB, CH, SAG contributed to drafting of the manuscript and read and approved the final manuscript.

## References

[B1] Cannon GA Carr MJ Yandle Z Schaffer K Kidney R Hosny G Doyle A Ryan J Gunson R Collins T Carman WF Connell J Hall WW A low density oligonucleotide microarray for the detection of viral and atypical bacterial respiratory pathogens J Virol Methods 2010 163 17 24 10.1016/j.jviromet.2009.07.005 19638287 PMC7112883

[B2] Felder KM Hoelzle K Wittenbrink MM Zeder M Ehricht R Hoelzle LE A DNA microarray facilitates the diagnosis of *Bacillus anthracis* in environmental samples Lett Appl Microbiol 2009 49 324 331 10.1111/j.1472-765X.2009.02664.x 19552771

[B3] Schmoock G Ehricht R Melzer F Rassbach A Scholz HC Neubauer H Sachse K Mota RA Saqib M Elschner M DNA microarray-based detection and identification of *Burkholderia mallei*, *Burkholderia pseudomallei* and *Burkholderia* spp Mol Cell Probes 2009 23 178 187 10.1016/j.mcp.2009.04.001 19366627

[B4] Järvinen AK Laakso S Piiparinen P Aittakorpi A Lindfors M Huopaniemi L Piiparinen H Mäki M Rapid identification of bacterial pathogens using a PCR- and microarray-based assay BMC Microbiol 2009 9 161 10.1186/1471-2180-9-161 19664269 PMC2741468

[B5] Wang D Coscoy L Zylberberg M Avila PC Boushey HA Ganem D DeRisi JL Microarray-based detection and genotyping of viral pathogens Proc Natl Acad Sci U S A 2002 99 15687 15692 10.1073/pnas.242579699 12429852 PMC137777

[B6] Chou CC Chen CH Lee TT Peck K Optimization of probe length and the number of probes per gene for optimal microarray analysis of gene expression Nucleic Acids Res 2004 32 e99 10.1093/nar/gnh099 15243142 PMC484198

[B7] Suzuki S Ono N Furusawa C Kashiwagi A Yomo T Experimental optimization of probe length to increase the sequence specificity of high-density oligonucleotide microarrays BMC Genomics 2007 8 373 10.1186/1471-2164-8-373 17939865 PMC2180184

[B8] Naiser T Ehler O Kayser J Mai T Michel W Ott A Impact of point-mutations on the affinity of surface-bound DNA/DNA and RNA/DNA oligonucleotide-duplexes: comparison of single base mismatches and base bulges BMC Biotechnol 2008 8 48 10.1186/1472-6750-8-48 18477387 PMC2435543

[B9] Karaman MW Groshen S Lee C-C Pike BL Hacia JG Comparisons of substitution, insertion and deletion probes for resequencing and mutational analysis using oligonucleotide microarrays Nucleic Acids Res 2005 33 e33 10.1093/nar/gni034 15722479 PMC549431

[B10] Southern E Mir K Shchepinov M Molecular interactions on microarrays Nat Genet 1999 21 5 9 9915493 10.1038/4429

[B11] Shchepinov MS Case-Green SC Southern EM Steric factors influencing hybridisation of nucleic acids to oligonucleotide arrays Nucleic Acids Res 1997 25 1155 1161 10.1093/nar/25.6.1155 9092624 PMC146580

[B12] Letowski J Brousseau R Masson L Designing better probes: effect of probe size, mismatch position and number on hybridization in DNA oligonucleotide microarrays J Microbiol Methods 2004 57 269 278 10.1016/j.mimet.2004.02.002 15063067

[B13] Kolchinsky A Mirzabekov A Analysis of SNPs and other genomic variations using gel-based chips Hum Mutat 2002 19 343 360 10.1002/humu.10077 11933189

[B14] Hadiwikarta WW Walter J-C Hooyberghs J Carlon E Probing hybridization parameters from microarray experiments: nearest-neighbour model and beyond Nucleic Acids Res 2012 40 e138 10.1093/nar/gks475 22661582 PMC3467032

[B15] Peplies J Glöckner FO Amann R Optimization strategies for DNA microarray-based detection of bacteria with 16S rRNA-targeting oligonucleotide probes Appl Environ Microbiol 2003 69 1397 1407 10.1128/AEM.69.3.1397-1407.2003 12620822 PMC150098

[B16] Wei T Pearson MN Armstrong K Blohm D Liu J Analysis of crucial factors resulting in microarray hybridization failure Mol Biosyst 2012 8 1325 1338 10.1039/c2mb05300d 22314967

[B17] Nguyen HK Southern EM Minimising the secondary structure of DNA targets by incorporation of a modified deoxynucleoside: implication for nucleic acid analysis by hybridisation Nucleic Acids Res 2000 28 3904 3909 10.1093/nar/28.20.3904 11024169 PMC110783

[B18] Peytavi R Tang LY Raymond FR Boissinot K Bissonnette L Boissinot M Picard FJ Huletsky A Ouellette M Bergeron MG Correlation between microarray DNA hybridization efficiency and the position of short capture probe on the target nucleic acid Biotechniques 2005 39 89 96 10.2144/05391RR01 16060373

[B19] Stedtfeld RD Wick LM Baushke SW Tourlousse DM Herzog AB Xia Y Rouillard JM Klappenbach JA Cole JR Gulari E Tiedje JM Hashsham SA Influence of dangling ends and surface-proximal tails of targets on probe-target duplex formation in 16S rRNA gene-based diagnostic arrays Appl Environ Microbiol 2007 73 380 389 10.1128/AEM.01785-06 17114322 PMC1796975

[B20] Guo Z Guilfoyle RA Thiel AJ Wang R Smith LM Direct fluorescence analysis of genetic polymorphisms by hybridization with oligonucleotide arrays on glass supports Nucleic Acids Res 1994 11 5456 5465 7816638 10.1093/nar/22.24.5456 PMC332096

[B21] Pierce KE Sanchez JA Rice JE Wangh LJ Linear-after-the-exponential (LATE)-PCR: primer design criteria for high yields of specific single-stranded DNA and improved real-time detection Proc Natl Acad Sci U S A 2005 102 8609 8614 10.1073/pnas.0501946102 15937116 PMC1150831

[B22] Tang X Morris SL Langone JJ Bockstahler LE Simple and effective method for generating single-stranded DNA targets and probes Biotechniques 2006 40 759 763 10.2144/000112154 16774119

[B23] Weller SA Cox V Essex-Lopresti A Hartley MG Parsons TM Rachwal PA Stapleton HL Lukaszewski RA Evaluation of two multiplex real-time PCR screening capabilities for the detection of *Bacillus anthracis*, *Francisella tularensis*, and *Yersinia pestis* in blood samples generated from murine infection models J Med Microbiol 2012 61 1546 1555 10.1099/jmm.0.049007-0 22899777

[B24] Janse I Bok JM Hamidjaja RA Hodemaekers HM van Rotterdam BJ Development and comparison of two assay formats for parallel detection of four biothreat pathogens by using suspension microarrays PLoS One 2012 7 e31958 10.1371/journal.pone.0031958 22355407 PMC3280232

[B25] Zhu LX Zhang ZW Liang D Jiang D Wang C Du N Zhang Q Mitchelson K Cheng J Multiplex asymmetric PCR-based oligonucleotide microarray for detection of drug resistance genes containing single mutations in *Enterobacteriaceae* Antimicrob Agents Chemother 2007 51 3707 3713 10.1128/AAC.01461-06 17646412 PMC2043267

[B26] Ballarini A Scalet G Kos M Cramer N Wiehlmann L Jousson O Molecular typing and epidemiological investigation of clinical populations of *Pseudomonas aeruginosa* using an oligonucleotide-microarray BMC Microbiol 2012 12 152 10.1186/1471-2180-12-152 22840192 PMC3431270

[B27] Urakawa H El Fantroussi S Smidt H Smoot JC Tribou EH Kelly JJ Noble PA Stahl DA Optimization of single-base-pair mismatch discrimination in oligonucleotide microarrays Appl Environ Microbiol 2003 69 2848 2856 10.1128/AEM.69.5.2848-2856.2003 12732557 PMC154504

[B28] Gresham D Dunham MJ Botstein D Comparing whole genomes using DNA microarrays Nat Rev Genet 2008 9 291 302 10.1038/nrg2335 18347592 PMC7097741

[B29] Ehricht R Slickers P Goellner S Hotzel H Sachse K Optimized DNA microarray assay allows detection and genotyping of single PCR-amplifiable target copies Mol Cell Probes 2006 20 60 63 10.1016/j.mcp.2005.09.003 16330186

